# Supplementary material for: The impact of top management team tenure heterogeneity on innovation efficiency of declining firms
Source: PLoS One. 2025 Feb 19;20(2):e0313624. doi: 10.1371/journal.pone.0313624 (PMC11838907; doi:10.1371/journal.pone.0313624)
Supplement: Attachment — Submitted filename: Comments_PONE-D-24-08354.docx [file pone.0313624.s002.docx]

**Abstract**

While the abstract effectively introduces the topic of the study and outlines the key factors under investigation, there is room for improvement in terms of providing more specific details about the methodology and findings. Enhancing the abstract with additional information on the study's contributions and practical implications could further engage readers and provide a more comprehensive overview of the research.

**Introduction**

The introduction could benefit from providing more context on the specific industry or region under study to help readers understand the applicability of the findings. Including information about the industry or industries represented in the sample could enhance the relevance of the study to practitioners in those sectors.

The transition from the general discussion of declining firms to the specific research objectives related to TMT tenure heterogeneity could be smoother.

**Literature Review and Hypotheses**

If the literature review heavily relies on secondary sources such as literature reviews and meta-analyses, there may be a risk of missing out on recent primary studies or unique perspectives. Incorporating a balance of primary and secondary sources can provide a more comprehensive understanding of the research domain.

The literature review and hypotheses section may be limited in scope, focusing primarily on TMT tenure heterogeneity and ownership concentration. Expanding the discussion to include related concepts or alternative explanations could enrich the theoretical framework and offer a broader perspective on the research topic.

If there are inconsistencies or conflicting findings in the literature, the section should address these discrepancies and provide a nuanced analysis of how the current study aims to contribute to resolving or clarifying such inconsistencies.

**The conclusion and implications**

The study's conclusions mainly focus on the immediate impact of TMT tenure heterogeneity and ownership concentration on innovation efficiency in declining firms. A weakness lies in the absence of a long-term perspective on how these factors may evolve over time and their sustained effects on the firm's innovation capabilities. Incorporating a longitudinal analysis could offer deeper insights into the dynamics of leadership and ownership structures in driving innovation outcomes.

The study's conclusions and implications are drawn from a specific context (A-share listed manufacturing firms in Shanghai and Shenzhen from 2015 to 2019). The lack of generalizability to firms in other industries or regions limits the broader applicability of the findings. Including a more diverse sample of firms or conducting comparative analyses across industries could enhance the study's relevance to a wider range of organizations.

While the study offers practical implications for declining firms, there is limited discussion on the potential challenges and barriers that organizations may face in implementing the recommended strategies. Addressing the practical hurdles associated with TMT changes, ownership restructuring, and innovation initiatives could provide a more realistic roadmap for firms seeking to improve their innovation efficiency.

The conclusion and implications section could benefit from exploring alternative solutions or strategies beyond TMT tenure heterogeneity and ownership concentration. Considering additional factors or approaches that could impact innovation efficiency in declining firms would enrich the practical insights offered and provide a more holistic view of the challenges and opportunities for organizational recovery.
